# Switchable Transducers in GaN MEMS Resonators: Performance Comparison and Analysis

**DOI:** 10.3390/mi12040461

**Published:** 2021-04-19

**Authors:** Imtiaz Ahmed, Dana Weinstein

**Affiliations:** Department of Electrical and Computer Engineering, Purdue University, West Lafayette, IN 47907, USA; danaw@purdue.edu

**Keywords:** MEMS resonator, GaN, heterostructure, 2DEG, switchable, piezoelectric, transducers, symmetric lamb mode, phonon trap cavity

## Abstract

This work presents a comprehensive comparison of switchable electromechanical transducers in an AlN/GaN heterostructure toward the goal of reconfigurable RF building blocks in next-generation ad hoc radios. The transducers’ inherent switching was achieved by depleting a 2D electron gas (2DEG) channel, allowing an RF signal launched by interdigital transducers (IDTs) to effectively excite the symmetric (S_o_) Lamb mode of vibration in the piezoelectric membrane. Different configurations for applying DC bias to the channel for electromechanical actuation in the piezoelectric are discussed. Complete suppression of the mechanical mode was achieved with the transducers in the OFF state. Equivalent circuit models were developed to extract parameters from measurements by fitting in both ON and OFF states. This is the first time that an extensive comparative study of the performance of different switchable transducers in their ON/OFF state is presented along with frequency scaling of the resonant mode. The switchable transducer with Ohmic IDTs and a Schottky control gate showed superior performance among the designs under consideration.

## 1. Introduction

Rapid evolution in wireless technology and increasing demand for high-bandwidth communication for 5G/6G and the Internet of Things (IoT) has necessitated a growing number of components in radio front-end modules in an increasingly overcrowded radio frequency (RF) spectrum. Low-cost ad hoc radios have drawn consumer interest, enabling new devices such as MEMS resonators for on-chip clocking (e.g., for massive MIMO), frequency-selective notch and passband filters, and spectral sensing due to their smaller footprint and low power consumption [1]. Additional losses associated with in-line solid-state or electromechanical RF switches in reconfigurable systems have spurred MEMS resonators integrated with out-of-line switches to improve the system’s overall noise figure and reduce system-level size and weight. Switchable RF filters based on electrostatic MEMS resonators provide inherent switching using the DC bias necessary for their linear actuation. However, these resonators and corresponding filters typically exhibit high insertion loss due to their low electromechanical coupling [2]. Intrinsically switchable piezoelectric MEMS resonators with high quality factor (Q) and coupling coefficient (k^2^) could provide a much-needed solution for compact, low-loss, tunable RF filters and oscillators in the GHz regime for wideband communication.

Gallium nitride (GaN) has been explored extensively as an electromechanical material due to its high coupling coefficient (k^2^~2%), acoustic velocity (~8000 ms^−1^), and low viscoelastic losses (f·Q~2.5 × 10^13^) that enable high-Q MEMS resonators with a scaling capability up to millimeter-wave frequencies [3,4,5,6,7,8]. GaN is also a wide bandgap semiconductor with high electron mobility and breakdown field, making it ideal for high-power and high-frequency applications in radio base stations and hand-held devices [9,10]. GaN high-speed electronics can also be integrated monolithically with MEMS resonators to suppress inherent parasitics, enabling a novel MEMS-IC platform for high-performance RF and millimeter-wave timing, spectrum monitoring, and configurable radios [11,12].

Mobile charges can be confined in a potential well created in GaN heterostructures (e.g., AlGaN/GaN, AlN/GaN) due to their large spontaneous and piezoelectric polarization [13]. The bias-dependent control mechanism of this 2D electron gas (2DEG) is traditionally used for the gate control of high electron mobility transistors (HEMTs) [14,15,16]. This same mechanism can be exploited to control electromechanical transducers in GaN-based MEMS [17,18].

GaN switchable transducers exciting bulk acoustic waves have been demonstrated in ref. [19], although they are limited by thickness-dependent resonance frequency. Devices with multiple frequencies on the same chip are possible, utilizing lithographically defined interdigital switchable transducers to launch and sense surface acoustic waves (SAWs) [20,21,22,23]. However, these are subject to high mechanical losses at high frequency and low acoustic velocity, which further limits their frequency scaling due to limits of lithographic resolution. Due to small frequency sensitivity to film thickness, high phase velocity and electromechanical coupling, switchable transducers integrated with GaN Lamb mode delay line filters have demonstrated low insertion loss, good out-of-band rejection, and high linearity [24]. Alternately, switchable transducers with 2DEG top and/or bottom electrodes were used to suppress the resonance mode [18,25,26,27], but they suffered from low loaded-Q due to the high resistance of the 2DEG compared to metal electrodes. Switchable resonators integrated with AlGaN/GaN HEMTs for sensing acoustoelectric signals have been demonstrated to design frequency references in oscillator circuits [28,29,30]. The actuation mechanism of normally OFF switchable transducers consists of piezoelectric and electrostatic forces caused by a depletion of 2DEG in the ON state, which results in significant improvement of the quality factor [31]. Actuation without a separate control gate for DC bias in such transducers has previously been demonstrated [31,32,33]. In our previous work, we have realized a switchable Lamb mode resonator with a Schottky control gate design showing improved transduction [34]. In this work, we perform a complete comparison and electromechanical analysis between various bias control mechanisms of normally OFF switchable transducers in GaN Lamb mode resonators.

## 2. Transducer Design

The switchable transducers in the GaN platform manipulate electromechanical fields to drive and sense RF MEMS resonators, utilizing bias dependence of the 2DEG in standard HEMT technology. Under 0 V bias, the high density of electrons in the 2DEG creates a conductive path that shields the electric field from penetrating the piezoelectric medium, resulting in a transducer OFF state. In the presence of particular applied bias, the 2DEG is depleted away from the channel that turns the transducers ON. The electric field generated by the RF signal at the IDTs can induce mechanical stress in the films. In this demonstration, the width and pitch of the IDTs are each designed to be one-quarter wavelengths of the zeroth-order symmetric (S_o_) Lamb mode. An AlN/GaN heterostructure was chosen over more common AlGaN/GaN; the former exhibits 2 × higher 2DEG concentration due to the wider bandgap of the AlN barrier layer [35]. However, these design concepts translate directly to alternative heterostructure stacks with high-density 2DEGs.

**Figure 1:**
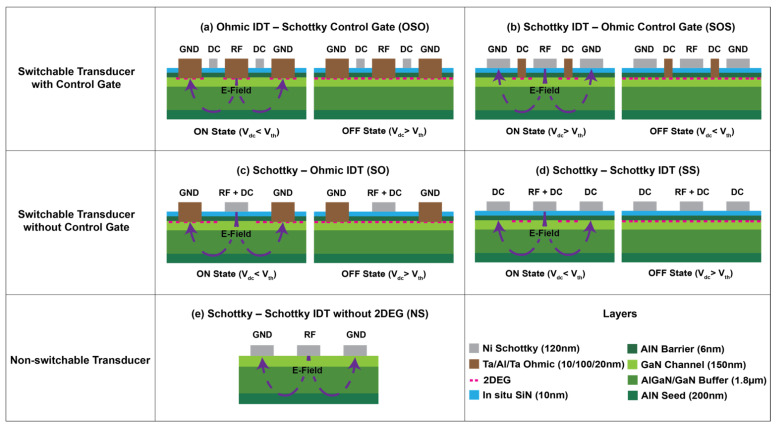
Schematic cross sections of different transducers considered in this work. Switchable transducer with (**a**) Ohmic–Ohmic IDT and Schottky control gate (OSO), (**b**) Schottky–Schottky IDT and Ohmic control gate (SOS), (**c**) Schottky–Ohmic IDT and no control gate (SO), (**d**) Schottky–Schottky IDT and no control gate (SS). (**e**) Non-switchable (NS) transducer with Schottky–Schottky IDT and no 2DEG channel.

Figure 1 shows a range of switchable transducer configurations investigated in this work in their ON/OFF states. They can be classified into two main categories depending on the method of DC bias application to switch the transducer states. (1) The switchable transducer design with a control gate has an independent gate meandering between IDTs to apply DC bias to deplete the 2DEG. It can be realized in two ways: Ohmic IDT–Schottky control gate (OSO) and Schottky IDT–Ohmic control gate (SOS). OSO devices (Figure 1a) need a negative DC bias below the threshold voltage to deplete the 2DEG from the channel. SOS devices (Figure 1b) require Schottky IDTs and Ohmic control gates to apply positive DC bias above the threshold voltage for efficient transduction.

(2) On the other hand, the switchable transducer design without a control gate shares the same IDTs for both DC bias and RF signal. It can be subdivided into two different configurations: Schottky–Ohmic IDT (SO) and Schottky–Schottky IDT (SS). In SO devices (Figure 1c), Schottky contacts are used to apply negative DC bias with respect to Ohmic contacts and carry the RF signal. In SS devices (Figure 1d), both polarities of the IDT are made of Schottky contacts and can apply negative DC bias below the threshold voltage to switch ON the transducer. For this case, 2DEG should be grounded using Ohmic contacts near the IDTs to efficiently deplete it away from the channel when DC bias is applied (not shown in Figure 1d). Non-switchable (NS) Lamb mode resonators with IDT composed of Schottky contacts were also considered in this work (Figure 1e). In NS devices, the AlN layer was removed to eliminate the 2DEG from the GaN structure.

## 3. Fabrication Process

Resonator fabrication was performed at Purdue’s Birck Nanotechnology Center using a commercial metal organic chemical vapor deposition (MOCVD) AlN/GaN heterostructure available from EpiGaN [36]. The heterostructure consisted of a thin AlN seed layer (200 nm) and alternating AlGaN/GaN buffer layers (1.8 µm) on a high resistivity (111) Si substrate for proper lattice matching and reduction in residual stress. A GaN channel (150 nm) and AlN barrier (6 nm) layers on top formed a potential well at their interface which confined a 2DEG at the heterojunction. Finally, a high-quality in situ SiN cap layer (10 nm) with a low density of pinholes ensured the stability of the 2DEG by passivating the sensitive surface from external damages during subsequent process steps.

**Figure 2:**
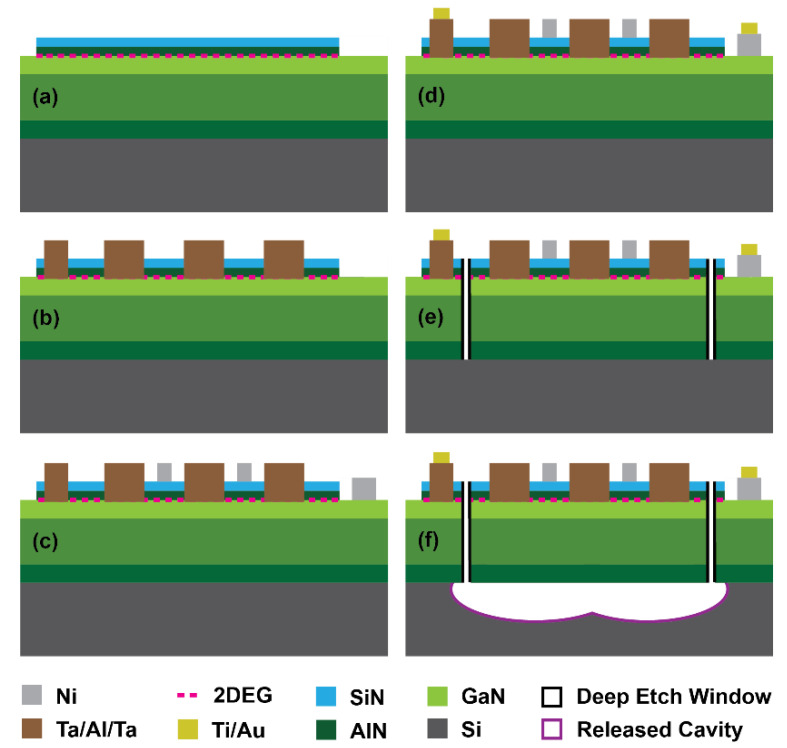
Fabrication process steps of switchable GaN MEMS resonators. (**a**) Mesa isolation. (**b**) Ohmic (Ta/Al/Ta) metallization and annealing. (**c**) Schottky (Ni) metallization. (**d**) Pad (Ti/Au) metallization. (**e**) Deep etch. (**f**) Isotropic release.

The fabrication process flow is shown in Figure 2. The process begins by selectively etching the AlN barrier layer using BCl_3_/Cl_2_ plasma using inductively coupled plasma–reactive ion etching (ICP-RIE) (Figure 2a). This defines the active device areas including the 2DEG for switchable transducers and provides electrical isolation between devices. Prior to that, in situ SiN is removed by CHF_3_/O_2_ ICP-RIE. The overall height of the Mesa islands is ~40 nm.

Au-free Ohmic contacts are essential for CMOS process compatibility [37], but are also important for high-Q acoustic resonators because Au causes additional losses through phonon–electron scattering and mass loading on the resonant cavities [28]. In this process, low resistance Au-free Ohmic contacts were achieved using a Ta(10 nm)/Al(100 nm)/Ta(20 nm) metal stack. The metals were deposited using an electron beam evaporator and then patterned with lift-off followed by rapid thermal annealing in an N_2_ ambient atmosphere at 575 °C for 10 min to drive the dopants into the 2DEG (Figure 2b). In situ SiN was removed initially to make an opening for metals by SF_6_ soft plasma using the same photoresist mask. Surface cleaning was performed using O_2_/Ar plasma followed by a HCl:H_2_O (1:3) dip before metal deposition. The obtained values of contact resistance and sheet carrier density for 2DEG from transmission line measurement (TLM) were 0.72 Ω·mm and 426 Ω/□, respectively. To create Schottky contacts, Ni(120 nm) was then evaporated and lifted off on the SiN cap (Figure 2c). Here, the SiN layer serves as a gate dielectric, which reduces gate leakage and improves gate control [38]. A shallow etch of the SiN cap was performed using buffered oxide etchant (BOE) to remove surface contamination before Ni deposition. Thick pads for landing probes and low resistance interconnections for IDTs and control gates were created by depositing and patterning Ti(10 nm)/Au(350 nm) away from acoustic cavities (Figure 2d).

The resonant cavities and release windows were defined with deep trenches (~2.2 µm) through AlN/GaN layers in a BCl_3_/Cl_2_ plasma in ICP-RIE to access the Si substrate (Figure 2e). An SPR220 photoresist mask was used to obtain a straight sidewall angle (~81°). The deep trench etch was optimized for AlN/GaN sidewall smoothness to ensure a high Q of the resonators. Finally, an isotropic etch of the Si was performed using XeF_2_ to release the resonators from the substrate (Figure 2f). A photoresist mask was used to protect the surface of the transducers from the etchant.

**Figure 3:**
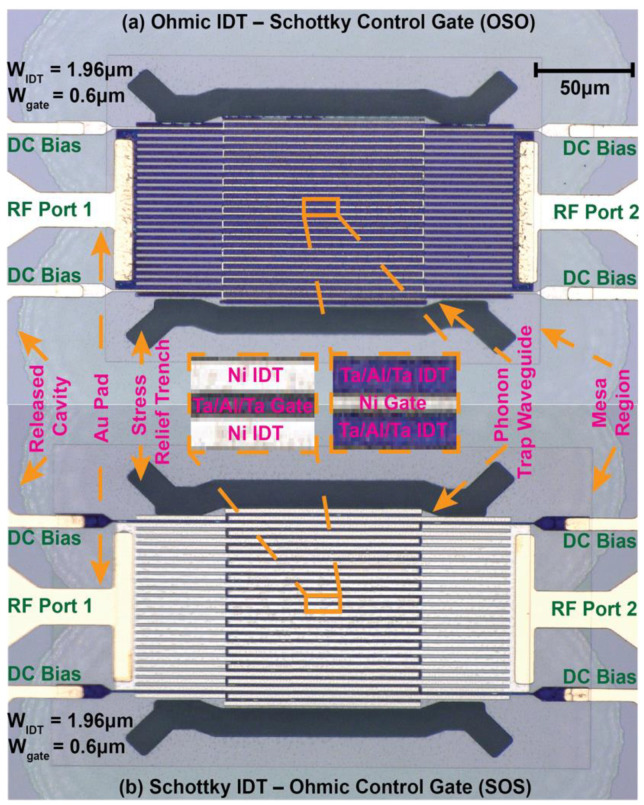
Optical micrographs of different switchable transducers with meandering control gates: (**a**) Ohmic IDT–Schottky control gate (OSO) and (**b**) Schottky IDT–Ohmic control gate (SOS). IDTs and control gates are shown in the inset.

Optical micrographs of two different switchable resonators with Ohmic IDT–Schottky control gate and Schottky IDT–Ohmic control gate are shown in Figure 3. Insets in the figure highlight the different types of IDTs and control gates of the transducers. In both cases, the devices have equal dimensions and numbers of IDTs. Identical phonon trap cavities with a wider active region in the middle and gradual reduction in width at the flanks are used to ensure high-Q resonance [32]. A standing elastic wave is formed in the resonance cavity, while the flanks generate an exponentially decaying evanescent wave to minimize radiative acoustic loss through the substrate [39,40]. The phonon trap design further eliminates the necessity of narrow tether suspensions and the resistive losses they induce. Additionally, a broad region to couple into the cavity improves power handling and linearity through better heat dissipation. Stress relief windows with curved design at the corners are also implemented to avoid fracture and increase device yield during release [5].

## 4. Results and Discussions

### 4.1. Measurement Setup

**Figure 4:**
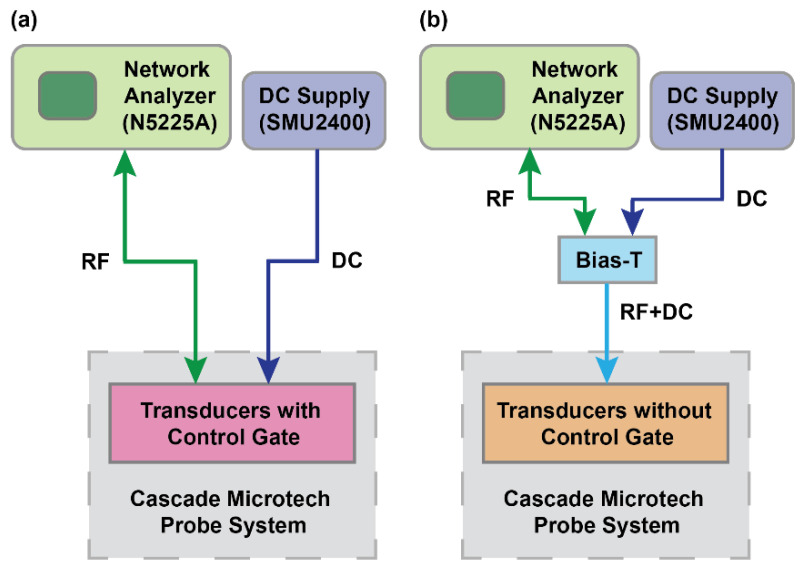
Schematic diagrams of the experimental measurement setup for switchable transducers (**a**) with a control gate and (**b**) without a control gate. In the case of transducers with a control gate, the RF signal was driven and sensed from IDTs and DC bias was applied to the control gates. On the other hand, combined RF and DC bias were applied to the IDTs using external bias-T for the transducers without a control gate.

The transducers were measured in a standard two-port configuration at room temperature under vacuum in a probe system (PMC200, Cascade Microtech, Dresden, Germany). A parametric network analyzer (N5225A, Agilent Technologies, Santa Clara, CA, USA) was used to drive −10 dBm input RF signal with 50 Ω termination, and scattering parameters (S-parameters) were extracted. DC bias was applied using source measurement units (SMU2400, Keithley Instruments, Solon, OH, USA) to control the transducers’ 2DEG channel. Measurements included short-open-load-through (SOLT) calibration using a standard calibration substrate to remove parasitics from the cables and probes. On-chip open and short structures were used to de-embed data from raw measurements to eliminate parasitic capacitance and inductance from the probe pads and routing. Two different experimental setups were used to measure switchable transducers, as shown in Figure 4. For the transducers with a control gate (OSO and SOS), RF signal and DC bias were applied to the IDTs and control gates, respectively. For resonators without a control gate (SS and SO), an external bias-T was used to superimpose the RF signal and DC voltage.

### 4.2. Transducers with Control Gate

**Figure 5:**
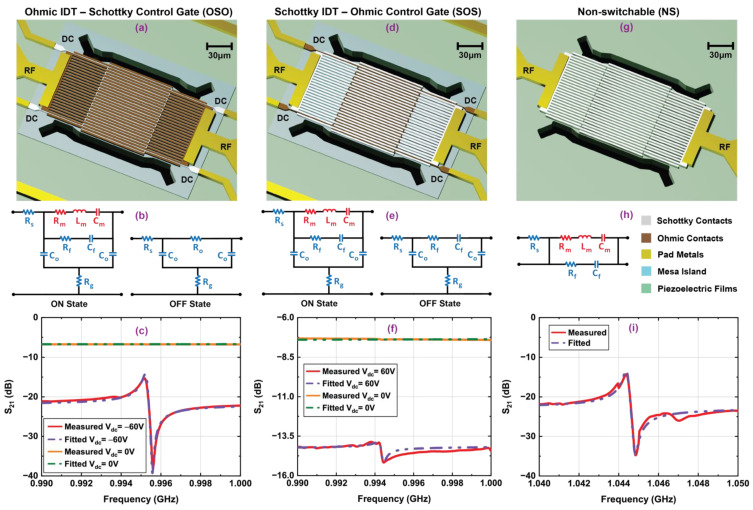
Three-dimensional schematic representation, equivalent circuit models and measured frequency responses along with fitted curves for switchable resonators: (**a**–**c**) Ohmic–Ohmic IDT with Schottky control gate (OSO), (**d**–**f**) Schottky–Schottky IDT with Ohmic control gate (SOS), and (**g**–**i**) non-switchable (NS) transducers. Both OSO and SOS have control gate width 0.6 µm, and all resonators have IDT width and pitch 1.96 µm. The devices were measured under vacuum with −10 dBm input RF power.

Figure 5 illustrates the 3D schematics, RF transmission measurements, and equivalent circuit models of OSO and SOS switchable transducers in their ON and OFF states. With zero DC bias, both transducers are in their OFF state because the 2DEG sheet carrier screens any field in the piezoelectric. The OSO device turns ON when the Schottky control gate’s voltage is lower than the negative threshold voltage of the channel, creating a depletion region under the gates and launching RF fields through the Ohmic IDTs into the piezoelectric materials beneath. On the other hand, efficient transduction in the SOS device needs positive DC bias above the threshold voltage at the Ohmic control gate, resulting in effective negative potential at the Schottky IDTs with respect to the channel and depleting 2DEG from the channel underneath the IDTs. Transmission measurements shown in Figure 5c,f demonstrate that both OSO and SOS transducers can generate the S_0_ Lamb mode at ~995 MHz when the DC biases at the control gates are −60 V and 60 V, respectively. The OFF-state characteristics of the transducers at zero bias are also shown in the same plots. The turn-ON voltages for both transducers are significantly high compared to the threshold voltage of on-chip HEMT (V_th_ = −6.4 V) shown in ref. [34]. This can be attributed to series resistance losses in the long meander control gates’ narrow electrical connections. A multi-layer metal process in which control gates bridge over the IDTs at every wavelength can be used to eliminate the problem.

To evaluate the performance of switchable transducers with control gates in their ON/OFF states, key metrics, i.e., mechanical and loaded quality factors (Q_m_, Q_l_), electromechanical coupling coefficient (k^2^), f·Q_m_ product, and figure of merit (FOM = k^2^·Q_l_) were determined by extracting parameters from lumped element equivalent circuit models and fitting with measured data using Advanced Design System (PathWave Design Software, Keysight Technologies, Santa Rosa, CA, USA). The extracted parameters are summarized in Table 1. For both switchable transducers in the ON state, a series R_m_–L_m_–C_m_ branch is used to model the resonator’s motional behavior, and R_f_–C_f_ are the electrical feedthrough components between the IDTs. The DC control gate acts as a small-signal ground for the RF ports and a physical capacitance (C_o_) between the IDT and gate needs to be included in the equivalent circuit models [41]. R_g_ and R_s_ are the resistances for the meander control gate and IDTs, respectively. Ohmic IDTs of the OSO transducers are shorted by the low-resistive 2DEG in the OFF state; therefore, the transducer’s mechanical branch is replaced by a small shunt resistance (R_o_) for the 2DEG and its contact with Ohmic IDTs. However, due to the in situ SiN and AlN barrier layer between Schottky IDTs and 2DEG in the SOS transducer in the OFF state, sizeable capacitive feedthrough between the RF ports suppresses mechanical modes. Both transducers exhibit an increase in transmission floor in the OFF state, as shown in Figure 5c,f. This bias-dependent floor shift due to direct electrical coupling between the drive and sense transducers may pose a challenge for RF front-end design. One solution is to design two separate resonant cavities for drive and sense transducers with control gates, and coupling them mechanically using suspended beams to allow strain fields to transfer acoustic energy at the resonance frequency. The improved electrical isolation between the drive and sense transducers in this scheme reduces feedthrough and ensures a constant broadband floor in the transmission spectra with respect to the DC bias at the control gates [28,33]. Larger electrical feedthrough due to high effective capacitance between Schottky IDTs for undepleted 2DEG and Ohmic control gates results in lower k^2^ in the ON state for SOS transducers relative to that of OSO devices. Meanwhile, phonon energy transferred to the sheet of electrons in the undepleted channel at maximum displacement points under the Ohmic control gates causes high acoustic losses and low Q_m_. As a result, the SOS transducer showed a decrease of 24% in the f·Q_m_ product and 56% in FOM compared to OSO.

A non-switchable transducer in which the 2DEG is removed from the channel and acoustic energy is confined in a highly resistive GaN buffer is ideal for comparison because the performances of normally OFF switchable transducers are mainly determined by the depletion of 2DEG and phonon–electron scattering in the channel [31]. The results from the on-chip NS transducer with identical dimensions, number of IDTs, and phonon trap cavity are shown in Figure 5g–i, and extracted parameters are summarized in Table 1. For consistency across this paper, C_f_ is used to denote the static capacitance for drive and feedthrough between IDTs, and R_f_ represents associated losses in the equivalent circuit model of the non-switchable transducer. Both OSO and SOS devices have a 5% lower resonance frequency than the NS device, which can be attributed to the higher mass loading of metal control gates and AlN/SiN layers in the Mesa island. The OSO transducer exhibits comparable ON-state f·Q_m_ product and FOM to that of the NS device, rendering it a superior transduction mechanism among the control gate designs explored in this work. This OFF-state shunt design could be suitable for fully tunable novel filter architecture, such as that shown in ref. [42].

### 4.3. Transducers without Control Gate

**Figure 6:**
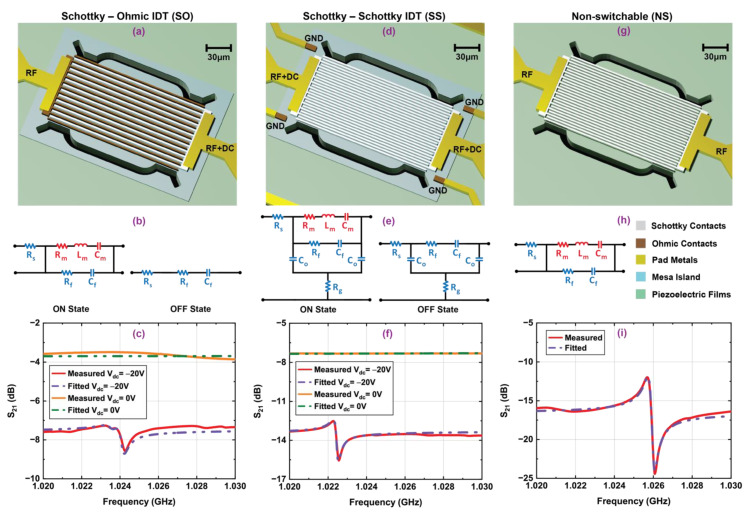
Three-dimensional schematic representation, equivalent circuit models, and measured frequency responses along with fitted curves for switchable transducers without control gates: (**a**–**c**) Schottky–Ohmic IDT (SO), (**d**–**f**) Schottky–Schottky IDT (SS), and (**g**–**i**) non-switchable (NS) transducers. All the resonators have IDT width and pitch 1.96 µm. The devices were measured under vacuum with −10 dBm input RF power.

A summary of the results obtained from switchable transducers without a control gate (SO and SS) along with a comparable non-switchable transducer is presented in Figure 6 and Table 2. It should be noted that this set of devices has a different radius of curvature in the phonon trap cavity compared to the design shown in Figure 5. This modification provides additional insight into the performance of the switchable transducers compared to the corresponding non-switchable device.

When a DC bias applied at the Schottky IDTs of the SO transducer is lower than the negative threshold voltage with respect to the Ohmic IDTs, the 2DEG is depleted from the channel and electromechanical transduction can effectively drive acoustic waves. On the other hand, the 2DEG in the SS transducer is grounded with Ohmic contacts, and both sets of Schottky IDTs require a negative DC bias lower than the threshold voltage to obtain superior transduction in the ON state [43]. Both transducers exhibit the S_0_ Lamb resonance at ~1.23 GHz at −20 V DC bias, shown in the frequency response plots in Figure 6c,f. The 2DEG is located under a thin AlN (6 nm) barrier layer; therefore, successful suppression of mechanical modes was experienced in both transducers, similarly to the transducers with the control gate. The equivalent circuit model for the SO transducer in its ON state contains a motional branch (R_m_–L_m_–C_m_) along with an electrical feedthrough branch (R_f_–C_f_) between IDTs and series resistance (R_s_) similar to the NS device. However, the SS transducer contains an undepleted 2DEG channel between Schottky IDTs in the ON state, because the 2DEG is only depleted under the Schottky contacts for moderate DC biases below threshold [21]. This results in additional capacitive feedthrough between Schottky IDTs and the undepleted 2DEG channel, which is represented by C_o_–R_g_ in the equivalent circuit model as shown in Figure 6e. It also increases phonon–electron scattering at the undepleted 2DEG channel due to the change in strain-induced deformation potential [44]. The combined effects of large capacitive feedthrough between Schottky IDTs and 2DEG channel along with phonon–electron scattering in the 2DEG result in a reduction in f·Q_m_ by 15% and FOM by 34% for SS resonator compared to the NS device. Acoustic impedance mismatch for different drive and sense IDT sets (Ni Schottky and Ta/Al/Ta Ohmic) is a likely reason for the additional spurious mode close to the primary resonance observed in the SO transducer. Acoustic energy leakage to the spurious mode significantly reduces the Q_m_ of the resonator. The SO devices have an overall reduction in f·Q_m_ by 46% and FOM by 52%. It is evident that the SO transducer should have higher feedthrough and phonon–electron scattering compared to the NS device due to undepleted 2DEG in the channel region between IDTs similar to the SS device noted earlier. However, it is difficult to quantitatively separate degradation in transducer performance from resonator performance due to the presence of the spurious mode. In both SO and SS resonators, the motional branch is suppressed by the capacitive feedthrough branch in the equivalent circuits for the OFF state.

### 4.4. Frequency Channelization

**Figure 7:**
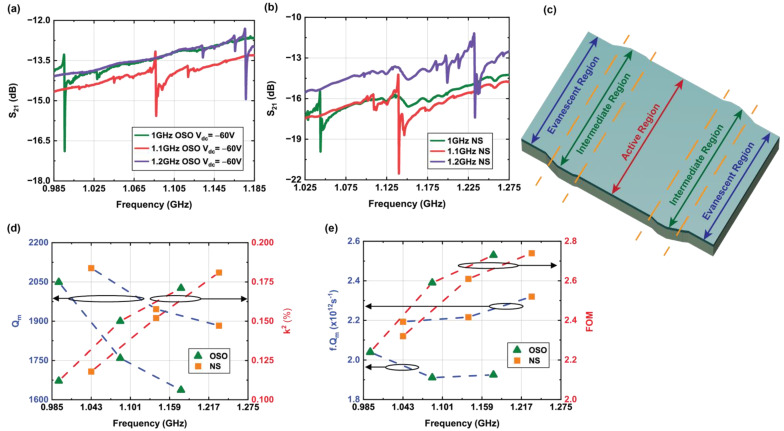
Transmission spectra of the resonators designed for different frequencies with (**a**) Ohmic IDT–Schottky control gate (OSO) switchable transducer at ON state and (**b**) non-switchable (NS) transducer. Width and pitch of the IDTs are scaled to have different resonant frequencies, but the number of IDTs and length of the phonon trap cavity are equal for all the transducers. (**c**) 3D schematic of phonon trap cavity with widened intermediate region between active and evanescent regions used for these transducers. Variation of (**d**) mechanical quality factor (Q_m_) and coupling coefficient (k^2^) along with (**e**) f·Q_m_ product and FOM with respect to frequency scaling for OSO and NS transducers.

Switchable resonators with OSO transducers and NS resonators were designed across a range of resonance frequencies (1/1.1/1.2 GHz) to demonstrate the channelization capabilities of this technology. Transmission characteristics of these devices are shown in Figure 7a,b. Due to fabrication yield, data are presented from resonators with a modified phonon trap cavity design shown in Figure 7c, which includes a widened intermediate region between the resonance cavity and the evanescent region connecting to the substrate. This phonon trap design exhibits ~2.5× lower Q_m_ and f·Q_m_ product relative to the direct-transition phonon trap designs of Figure 5 and Figure 6. Nonetheless, this frequency band demonstration interrogates the performance of the switchable GaN resonators across frequency.

The width and pitch of the IDTs for the resonators are scaled to actuate the S_0_ Lamb mode at 1, 1.1, 1.2 GHz. All devices share the same number of IDTs and cavity length. A comparison in Q_m_ and k^2^ with frequency scaling for OSO and NS transducers is shown in Figure 7d. Q_m_ reduces while k^2^ increases with the frequency for both OSO and NS transducers in a similar trend. Therefore, the f·Q_m_ product remains almost constant, and the FOM increases gradually with the frequency, as presented in Figure 7e.

A commercially available AlN/GaN-on-Si wafer used in this work was designed for a high-concentration 2DEG at the heterointerface for HEMT technology, and GaN/AlGaN/AlN layers were introduced in the buffer region to reduce residual stress during high-temperature MOCVD growth. Increased acoustic scattering in defects at the interfaces of these layers along with mass loading in the metal electrodes are possible reasons for the lower f·Q_m_ product obtained in this work, compared to the theoretical Akhiezer limit of phonon–phonon scattering for AlN(~10^13^)/GaN(~2.5 × 10^13^) [3]. The increasing trend of k^2^ with frequency shown in Figure 7d matches qualitatively with the simulated intrinsic coupling coefficient for the S_0_ Lamb mode in GaN, corresponding to the thickness-to-wavelength ratio derived in ref. [45], although crystal imperfections and mass loading due to metals reduce k^2^ values obtained from experimental results. Meanwhile, only top electrodes are used to launch the S_0_ Lamb mode in the resonators to avoid fabrication complexity. By choosing the correct thickness-to-wavelength ratio and adding bottom electrodes, k^2^ can be increased by ~4× for these transducers [46].

## 5. Conclusions

Switchable MEMS resonators in GaN MMIC technology with different control mechanisms of transduction have been experimentally demonstrated on an AlN/GaN heterostructure. Inherent switching in the transducers was achieved by applying a DC bias to selectively deplete the 2DEG at the heterointerface. The transducers exhibited excellent suppression of the mechanical mode in the OFF state. A comparative study of different switchable transduction mechanisms and non-switchable devices is discussed, with experimental verification of the Ohmic IDT–Schottky control gate transduction mechanism exhibiting the highest f·Q_m_ product and FOM in the ON state. Effects of frequency scaling on transducer performance were analyzed in the narrow range of interest for channel-select capabilities. In the future, these transducer designs could be extended to high k^2^ material platforms such as AlScN with III–V HEMT layers [47,48] to provide high-performance RF components for programmable radios for real-time spectrum sensing.

## Figures and Tables

**Table 1:** Fitting parameters of the switchable transducers with control gate in ON/OFF states along with their non-switchable counterparts extracted from equivalent circuit models shown in Figure 5. Color coding coincides with the circuits shown in Figure 5.

Device	State	V_dc_ (V)	f_s_ (GHz)	R_m_ (Ω)	L_m_ (µH)	C_m_ (aF)	C_f_ (fF)	R_f_ (Ω)	C_o_ (fF)	R_o_ (Ω)	R_g_ (Ω)	R_s_ (Ω)	Q_m_	Q_l_	k^2^ (%)	f·Q_m_ (×10^12^)	FOM
OSO	ON	−60	0.995	351	284	90	75.8	35.5	246.5	-	253	27.4	5067	4700	0.11	5.04	5.18
	OFF	0	-	-	-	-	-	-	3060	80.5	253	27.4	-	-	-	-	-
SOS	ON	60	0.994	2780	1707	15	302	32.1	345.0	-	190	53.1	3837	3765	0.06	3.82	2.26
	OFF	0	-	-	-	-	352	41.2	2195	-	190	53.1	-	-	-	-	-
NS	-	-	1.044	345	276	84	111	40.5	-	-	-	53.4	5248	4546	0.12	5.48	5.46

**Table 2:** Fitting parameters of the switchable transducers without control gate in ON/OFF states along with their non-switchable counterparts extracted from equivalent circuit models shown in Figure 6. Color coding coincides with the circuits shown in Figure 6.

Device	State	V_dc_ (V)	f_s_ (GHz)	R_m_ (Ω)	L_m_ (µH)	C_m_ (aF)	C_f_ (fF)	R_f_ (Ω)	C_o_ (fF)	R_o_ (Ω)	R_g_ (Ω)	R_s_ (Ω)	Q_m_	Q_l_	k^2^ (%)	f·Q_m_ (×10^12^)	FOM
SO	ON	−20	1.023	3550	1099	22	840	11.9	-	-	-	37.9	1990	1969	0.04	2.04	0.79
			1.024	861	335	72	840	11.9	-	-	-	37.9	2507	2401	0.07	2.57	1.68
	OFF	0	-	-	-	-	3615	8.9	-	-	-	37.9	-	-	-	-	-
SS	ON	−20	1.022	1231	757	32	277	12.9	206	-	850	45.9	3951	3809	0.06	4.03	2.29
	OFF	0	-	-	-	-	303	15.9	1195	-	850	45.9	-	-	-	-	-
NS	-	-	1.026	331	236	102	239	23.5	-	-	-	63.9	4605	3858	0.09	4.72	3.48

## Data Availability

Data are available upon request from the corresponding author.
